# A GMMA-CPS-Based Vaccine for Non-Typhoidal *Salmonella*

**DOI:** 10.3390/vaccines9020165

**Published:** 2021-02-17

**Authors:** Akosiererem S. Sokaribo, Sumudu R. Perera, Zoe Sereggela, Ryan Krochak, Lindsay R. Balezantis, Xiaohui Xing, Shirley Lam, William Deck, Sam Attah-Poku, Dennis Wade Abbott, Shantanu Tamuly, Aaron P. White

**Affiliations:** 1 Vaccine and Infectious Disease Organization-International Vaccine Centre, Saskatoon, SK S7N5E3, Canada; ats172@mail.usask.ca (A.S.S.); sumudu.perera@usask.ca (S.R.P.); zhs569@mail.usask.ca (Z.S.); rdk945@mail.usask.ca (R.K.); lindsay.balezantis@usask.ca (L.R.B.); shirley.lam@usask.ca (S.L.); will.deck@usask.ca (W.D.); sam.attah-poku@usask.ca (S.A.-P.); 2 Department of Biochemistry, Microbiology and Immunology, University of Saskatchewan, Saskatoon, SK S7N5E5, Canada; 3 Agriculture and Agri-Food Canada, Lethbridge Research and Development Centre, Lethbridge, AB T1J4B1, Canada; xiaohui.xing@canada.ca (X.X.); wade.abbott@canada.ca (D.W.A.); 4 Department of Veterinary Biochemistry, College of Veterinary Science, Assam Agricultural University, Khanapara, Guwahati 781022, Assam, India; shantanu.tamuly@aau.ac.in

**Keywords:** vaccines, *Salmonella*, capsular polysaccharide, colanic acid, GMMAs

## Abstract

Non-typhoidal *Salmonella* are a major cause of gastroenteritis worldwide, as well as causing bloodstream infections in sub-Saharan Africa with a high fatality rate. No vaccine is currently available for human use. Current vaccine development strategies are focused on capsular polysaccharides (CPS) present on the surface of non-typhoidal *Salmonella*. This study aimed to boost the amount of CPS purified from *S. Typhimurium* for immunization trials. Random mutagenesis with Tn*10* transposon increased the production of CPS colanic acid, by 10-fold compared to wildtype. Immunization with colanic acid or colanic acid conjugated to truncated glycoprotein D or inactivated diphtheria toxin did not induce a protective immune response in mice. However, immunization with Generalized Modules for Membrane Antigens (GMMAs) isolated from colanic acid overproducing isolates reduced *Salmonella* colonization in mice. Our results support the development of a GMMA-CPS-based vaccine against non-typhoidal *Salmonella*.

## 1. Introduction

*Salmonella enterica* species are the major cause of foodborne diseases, typhoid fever and gastroenteritis [1]. Typhoid fever, common in developing countries, is mainly caused by typhoidal *Salmonella* (TS) serovars: *S*. Typhi and *S.* Paratyphi [2]. Gastroenteritis occurs worldwide and is primarily caused by non-typhoidal *Salmonella* (NTS) serovars: *S*. Typhimurium and *S.* Enteritidis [2]. The global incidence of NTS gastroenteritis is estimated at ~94 million cases annually, with 155,000 deaths, and ~80.3 million cases are contracted via foodborne transmissions [3]. NTS gastroenteritis is usually self-limiting in immunocompetent individuals and severe cases can be treated with antibiotics. However, there is an increase in multi-drug-resistant isolates [4], and in sub-Saharan Africa, NTS serovars associated with gastroenteritis globally are the leading cause of bloodstream infections that are distinct from typhoid fever [5,6]. A protective vaccine against NTS serovars and implementation of effective food safety procedures are needed to reduce the global incidence of gastroenteritis.

Capsular polysaccharides (CPS) are carbohydrate-based polymers synthesized and expressed on bacterial surfaces [7]. The CPS that have been identified in NTS are colanic acid, lipopolysaccharide (LPS) and O-Ag capsule [8,9,10]. Colanic acid composed of repeating units of glucose, galactose and fucose [8] is produced at temperatures below 30 °C and is thought to be involved in the survival of adverse environmental conditions [11,12]. LPS is the major component of the outer membrane of Gram-negative bacteria. It plays an important role in bacterial pathogenesis, and in the stimulation of an immune response [10]. O-Ag capsule is a group IV CPS that has been implicated in *Salmonella* resistance to desiccation and killing by human immune serum [9,13]. Due to their surface location, CPS stimulate immune responses to bacteria and are optimal targets for vaccine development.

The LPS O-Ag is the target of protective immunity against NTS, however, the immune response is strain- or serovar-specific [14,15,16]. Since there are >1500 NTS serovars, a vaccine that can induce cross-protective immunity against the predominant NTS serovars will reduce the prevalence of gastroenteritis. The O-Ag capsule is an attractive vaccine candidate because it is comprised of tetrasaccharide repeating units of mannose, rhamnose, galactose and tyvelose or abequose, similar to LPS O-Ag [9,17]. Despite their similar repeating units, O-Ag capsule differs from LPS O-Ag in several ways: [1] O-Ag capsule is comprised of >2000 repeat units, while LPS O-Ag is comprised of <100 repeating units, [2] the tyvelose or abequose sugar in O-Ag capsule is glycosylated, [3] O-Ag capsule has a lower net charge and (4) O-Ag capsule is not associated with the LPS core region [9,17]. The increased molecular weight of oligosaccharides has been shown to result in improved immune response [18], and glycoconjugates from high molecular weight polysaccharides are immunogenic in humans at lower doses [19]. Due to the large number of repeating units, we hypothesized that immune response to O-Ag capsule could induce cross-protective immunity against the predominant NTS serovars that cause gastroenteritis.

Outer membrane vesicles (OMVs) are spherical outer membrane blebs spontaneously released by Gram-negative bacteria [20]. They are comprised of proteins, CPS and lipoproteins present on the outer membrane, and are released in all growth phases, and during infection [20,21]. OMVs are attractive vaccine candidates because they present antigens in their natural context to the immune system [20]. However, the amount of spontaneously released OMVs is not enough for vaccine development. Genetic modifications that disrupt the integrity of the inner and outer membrane have been shown to enhance OMV production [22,23]. OMVs released from genetically modified bacteria are called Generalized Modules for Membrane Antigens (GMMAs) [24]. GMMAs are desirable targets for the development of subunit vaccines because they lack the ability to cause disease, can deliver heterologous antigens [25,26] and are immunogenic [27]. Proteomic analysis has shown that GMMAs contain over 100 outer membrane and periplasmic proteins [24,28]. Due to the large number of antigens that GMMAs can present to the immune systems, GMMAs are being exploited for the development of cross-protective vaccines, for diseases like gastroenteritis caused by several NTS serovars [29].

In this study, we describe conditions that improve CPS production in *S*. *Typhimurium*. We also show how random mutagenesis aimed at increasing the production of O-Ag capsules for vaccine development led to increased colanic acid production. Immunization with colanic acid did not induce a protective immune response against *S*. *Typhimurium*. However, immunization with GMMAs purified from colanic acid overproducing *Salmonella* reduced bacterial colonization in mice.

## 2. Materials and Methods

### 2.1. Bacterial Strains, Media and Growth Conditions

The bacterial strains used in this study are listed in Table 1. For standard growth, strains were inoculated from frozen stocks onto LB agar (lysogeny broth, 1% NaCl, 1.5% agar) supplemented with an appropriate antibiotic (50 µg/mL kanamycin (Kan), 34 µg/mL chloramphenicol (Cam) or 5 µg/mL tetracycline (Tet)) and grown overnight at 37 °C. Isolated colonies were used to inoculate 5 mL LB broth and the culture was incubated for 18 hours at 37 °C with agitation at 200 rpm. For EPS purification, overnight cultures were grown for 5 days at 28 °C on agar supplemented with 1% glucose, 0.05% yeast extract, 10 mM Na_2_HPO_4_, 0.1% NH_4_Cl, 0.3% KH_2_PO_4_, with or without 40 mM MOPS (3-(N-morpholino)propanesulfonic acid). For murine infection experiments, overnight cultures grown in LB broth were diluted to the desired CFU concentration in 100 mM HEPES (4-(2-hydroxyethyl)-1-piperazineethanesulfonic acid), pH 8, and used to infect mice.

For bioluminescence assays, overnight cultures of *S*. *Typhimurium* transformed with promoter luciferase fusions (*yihUTSRQPO*::*luxCDABE* or *yihVW*::*luxCDABE*), were diluted 1 in 600 in 1% tryptone broth supplemented with 50 µg/mL Kan to a final volume of 150 µL in 96-well clear-bottom black plates (9520 costar Corning Inc) and overlaid with 50 µL of mineral oil. Cultures were assayed for luminescence (1 s; in counts per second (cps)) and absorbance (590 nm, 0.1 s) every 30 min during growth at 28 °C, with agitation in a Victor X3 multilabel plate reader (Perkin-Elmer, Waltham, MS, USA).

### 2.2. Generation of S. *Typhimurium* Mutant Strains

The lambda red recombinase knockout procedure [34] was used to generate *S*. Typhimurium Δ*lon*, Δ*tolR*, Δ*yihW*, ∆*yih* and Δ*yihVW* mutants. Primers containing 50-nucleotide sequences on either side of *lon*, *tolR*, *yih*, *yihW* or *yihVW* (Table 2) were used to amplify the *cat* gene from pKD3 or *kan* gene from pKD13 using Phusion High-Fidelity DNA polymerase (New England Biolabs, Ipswich, MS, USA). The PCR products were purified and electroporated into *S*. Typhimurium 14028 cells containing pKD46. Mutants were selected by growth at 37 °C on LB agar supplemented with 10 µg/mL Cam or 50 µg/mL Kan. Cam^R^ isolates were re-streaked onto LB agar containing 34 µg/mL Cam. Mutations were transduced into *S*. Typhimurium strains with a clean background using P22 phage [35]. The *cat* or *kan* gene was resolved from the chromosome using pCP20 [34]. DNA sequencing of PCR products from the chromosomes of mutant *Salmonella* isolates was used to verify the loss of *lon*, *tolR*, *yih*, *yihW* or *yihVW*. Primers used in this study are listed in Table 2.

### 2.3. Generation of Transposon Mutants

To generate *S*. Typhimurium Δ*bcsA* Δ*yihW* transposon mutants [30,32], *S*. Typhimurium LT2 harboring pNK972 was infected with P22 lysate of *S*. Typhimurium TT10423 containing Tn10dtet on F’. The resulting transductants (~100,000 colonies) were pooled and a P22 *S*. Typhimurium LT2 Tn10dtet transducing fragment library was generated, following the method outlined by Maloy [35]. The resulting P22 phage lysate was used to infect *S*. Typhimurium Δ*bcsA* Δ*yihW* and plated on 1% tryptone supplemented with 10 µg/mL Tet. The resulting transductants (10,000 to 20,000) were visually screened for the presence of a mucoid morphology, which was indicative of high CPS production. To identify the site of transposon insertion, nested PCR was performed on purified genomic DNA using primers TL or TR and ARB1 or ARB6. The product of the first reaction was further amplified using primers UniversalTn and ARB2. PCR products were purified and sequenced. To identify the site of Tn*10* insertion, DNA sequence was mapped to *S*. Typhimurium 14028 using Geneious 9.0.5 (https://www.geneious.com)

### 2.4. Generation of Plasmid Vectors

To generate pBR322-*yihVW*, the DNA region containing *yihVW* was PCR-amplified from *S*. Typhimurium 14028 genomic DNA using primers yihVWFOR and yihVWREV (Table 2) and Phusion polymerase (Thermo Fisher Scientific, Waltham, MS, USA), following reaction conditions as recommended by the manufacturer. The resulting PCR product was digested with *AatII* and *PstI* and ligated into *AatII*/*PstI*-digested pBR322 prior to electroporation into *S*. Typhimurium 14028. Positive clones were selected by growth on LB agar supplemented with 7 ug mL^−1^ Tet. The generation of pBR322-*yihVW* plasmid was confirmed by sequencing using primers yihVWseqF and yihVWseqR (Table 2). The generation of *yihUTSRQPO* and *yihVW* promoter region fused to *luxCDABE* operon has previously been described [9].

### 2.5. Purification and Neutral Monosaccharide Analysis of Capsular Polysaccharide

The capsular polysaccharide (CPS) purification protocol was adopted from Gibson et al. [9] with some modifications. To separate EPS from other cellular materials, *S.* Typhimurium cells scraped off the agar surface were resuspended in 1% phenol, mixed vigorously by vortexing and sedimented by centrifugation (16,000× *g*, 4 °C, 4 h). Crude EPS was precipitated from the supernatant by adding 4 volumes of ice-cold acetone while stirring continuously with a glass rod. The resulting precipitate was stored overnight at −20 °C to allow for further precipitation, sedimented by centrifugation (6000× *g,* 4 °C, 15 min) and air-dried. Crude EPS was resuspended in water, dialyzed in water for 48 h (MW cut-off 10 kDa; SnakeSkin dialysis tubing, Thermo Fisher Scientific) and lyophilized. The lyophilized polysaccharide was dissolved in buffer A (15 mM NaOAc, 0.05% Triton X-100, pH 5.5) and 0.01% sodium azide. The dissolved sample was heated twice at 37 °C for 15 min prior to loading onto Q Sepharose FF xk50/11.5 and washing with 2 column volumes of buffer A. Materials were eluted with a stepwise gradient of buffer B (1.5 M NaOAc, 0.05% Triton X-100, pH 5.5), which was increased sequentially from 17% (1.25 column volumes) to 50% (1.25 column volumes) to 100% (1.5 column volumes). CPS-specific serum was used to identify fractions containing cross-reactive material by western blot.

For size exclusion chromatography, colanic acid containing fractions were pooled, concentrated (10 MWCO centrifuge filters, Amipore) and filtered through a 0.22 µm syringe tip filter, prior to loading on to the Superdex S300 prep grad xk26/95 column. The column was washed with 50 mM NH_4_HCO_2_ pH 7.72. Colanic acid containing fractions were identified using Western blot and concentrated with a Millipore Amicon Ultra-15 Centrifugal Filter Device, dialyzed in water for 48 h (MW cutoff 10 kDa; SnakeSkin dialysis tubing; Thermo Fisher Scientific) and lyophilized.

The dry polysaccharide samples were treated by thermostable α-amylase and α-glucosidase, followed by extensive dialysis against de-ionized water and freeze-drying to make sure the samples were glycogen-free [36]. The lyophilized samples were then hydrolyzed to monosaccharides by trifluoacetic acid, reduced with sodium borohydride, and acetylated using acetic anhydride [37]. The resulting alditol acetates were analyzed on an Agilent HP 5890 Series II gas chromatograph coupled to a flame ionization detector (GC-FID) fitted with a Rtx-2330 capillary column (15 m length × 0.32 mm internal diameter × 0.2 μm film thickness; Restek Corporation). Inlet temperature was kept consistently at 250 °C and an injection volume of 1 µL was used for all injections. For each sample run, the oven temperature was programmed to hold at 180 °C for 2 min, increase from 180 to 230 °C at 4 °C/min, from 230 to 260 °C at 8°C/min, followed by holding at 260 °C for 10 min, and the inlet program was programmed to increase from 5.0 to 6.0 psi at 0.10 psi/min, from 6.0 to 7.0 psi at 0.20 psi/min and from 7.0 to 8.0 psi at 0.40 psi/min, followed by holding at 8.0 psi for 10.75 min (total run time 28.25 min). Relative monosaccharide composition (mol%) was calculated by referring to the FID responses of monosaccharide standards. Experiments were conducted in triplicate.

### 2.6. Endotoxin Removal from Purified EPS

For endotoxin removal by acid hydrolysis, purified crude EPS were dissolved in 1% acetic acid and heated in an oil bath at 110 °C for 100 min [38]. The solution was centrifuged for 10 min at 8000 rpm. Pellet was discarded and the supernatant was lyophilized before colanic acid isolation using anion exchange and size exclusion chromatography.

For endotoxin removal using Triton X-114, purified colanic acid was dissolved in pre-condensed Triton X-114, prepared by dissolving 20 mL of Triton X-114 and 16 mg of 2, 6-Di-tert-butyl-4-methylphenol at 4 °C in 10 mM Tris-HCl (pH 7.4), 150 mM NaCl solution [39]. The mixture was incubated at 30 °C until it separated into a detergent-depleted aqueous phase and a detergent-rich phase. The large aqueous phase was discarded and replaced with an equal volume of 10 mM Tris-HCl (pH 7.4), 150 mM NaCl. The solution was mixed and incubated at 30 °C to enable phase separation. After three rounds of phase separation, the detergent-rich phase with a Triton X-114 concentration of 11%, was collected and stored at room temperature [38].

For endotoxin removal using pre-condensed Triton X-114, purified EPS was dissolved in water to a final concentration of 0.5 mg/mL and mixed with 11% Triton X-114 to a final Triton X-114 concentration of 1% (*w*/*v*). The resulting cloudy solution was stirred for 30 min at 4 °C until the solution became clear. The mixture was incubated at 37 °C for 30 min to induce separation into two phases, and subsequently centrifuged at 1200× *g* for 30 min at 25 °C. The lower phase containing LPS was discarded and the upper phase containing EPS was mixed with 11% Triton X-114 to a final concentration of 2%. LPS extraction was repeated twice as described above. Centrifugation speed and time were increased by 1000× *g* and 30 min for each round of purification to obtain the best separation.

To remove detergent from the EPS-containing solution after LPS removal, the Triton X-114-treated samples were mixed with three volumes of methanol–chloroform (two volumes of methanol and one volume of chloroform) and incubated at room temperature for 30 min to enable phase separation. The Triton X-114 containing lower phase was eluted and the colanic acid containing upper phase was mixed again with the methanol–chloroform solution. This procedure was repeated two additional times. Residual methanol–chloroform was removed using water aspiration. Samples were dialyzed for 48 h in ddH_2_O at 4 °C and lyophilized.

### 2.7. Conjugation of EPS to Truncated Glycoprotein D (tgD) and CRM197

Twenty mg of purified colanic acid dissolved in 0.15 M NaCl (2 mL final volume) was allowed to react with 13 mg of sodium cyanoborohydride (CNBr, dissolved in 16 µL chloroform), and the pH of the solution was maintained at pH 10.5 using NaOH (0.5 M). After 15 min at room temperature, adipic dihydrazide (ADH) was added to a final concentration of 12 mg/mL. HCl (0.5 M) was used to reduce the final pH to 8.5. The reaction mixture was incubated at room temperature overnight with stirring. The solution was dialyzed twice against 20 mM MES buffer (pH 6.0) using a 10 kDa cut-off MWCO dialysis tubing for 3 hours at 4 °C [40]. Inactivated diphtheria toxin CRM197 was obtained from Fina Biosolutions LLC (Rockville, MD, USA). The purification of truncated glycoprotein has been previously described [41].

The resulting CNBr-treated CPS (5 mg/mL, 3 mL) was allowed to react with 1.5 mL tGD or CRM197 (2 mg/mL) in the presence of 12 mg EDC (dissolved in 20 mM MES buffer pH 6.0) at room temperature overnight. The resulting solution was dialyzed against 0.15 M NaCl using 10 kDa MWCO dialysis tubing for 3 h at 4 °C. CPS conjugated to tGD (CA-tgD) or CRM197 (CA-CRM197) was loaded onto a Sephacryl S300 xk26/94 column. The column was washed with 50 mM PBS, 15 mM NaCl, pH 7.4 solution and 10 mL fractions were collected. Fractions containing conjugated material were identified based on size after western blot analysis with EPS-specific serum.

### 2.8. Outer Membrane Vesicles (GMMAs) Production and Purification

For GMMA production, bacteria grown overnight at 37 °C were used to inoculate 1% tryptone broth (without antibiotics) to an optical density of 0.03 at 600 nm. Cultures were incubated at 30 °C, for 18 h with agitation (200 rpm). Culture supernatants were collected by centrifugation at 8000× *g* for 10 min, filtrated with a 0.45 µm filter, and concentrated by dialysis (MW cut-off 10 kDa; SnakeSkin dialysis tubing, Thermo Fisher Scientific) against polyethylene glycol (PEG) 2000. GMMAs were pelleted by ultra-centrifugation at 186,000× *g* for 2 h at 4 °C. The resulting pellets were resuspended in endotoxin-free water, filtered with a 0.22 µm filter, lyophilized, and resuspended in PBS.

### 2.9. Vaccine Formulation

Vaccine antigens, colanic acid (CA), CA-tgD and CA-CRM197 were formulated with triple combination adjuvant consisting of Polyinosinic:polycytidylic acid (Poly (I:C)) (Thermo Fisher Scientific), host defense peptide (HDP) and polyphosphazene (PCEP) (Idaho National laboratory) immediately prior to administration. Formulations were prepared by first mixing 10 µg of PIC and 20 µg HDP in PBS and incubating at room temperature (RT) for 15 min. CA, CA-tgD or CA-CRM197 were added before the addition of 10 µg of PCEP, making a final ratio of 1:2:1 of PIC:HDP:PCEP (TriAdj). Mixtures were incubated in the dark for 15 min at RT prior to administration.

### 2.10. Murine Immunization Experiments

Six- to eight-week-old female BALB/c mice were purchased from Charles River Laboratories (Kingston, ON), while C57BL/6 mice were purchased from Jackson Laboratory (Bar Harbor, ME). Mice were assigned to cage groups using a randomization table prepared in Microsoft Excel and individual mice were marked with ear notches.

Two groups of six BALB/C mice were immunized intramuscularly 3 times at 2-week intervals with 50 µg colanic acid (CA), 1 µg CA conjugated to tGD (CA-tGD), or phosphate-buffered saline (PBS). Serum was collected on days 0, 15, 29, 43 and 58. Three groups of six C57BL/6 mice were immunized intramuscularly 2 times at 3-week intervals with 50 µg CA, 50 µg CA conjugated inactivated diphtheria toxin (CA-CRM197), or PBS. Serum was collected on days 0, 21, 42 and 63.

For immunization with GMMAs, four groups of six C57BL/6 mice were immunized intramuscularly with PBS or 50 µg of GMMAs purified from *S*. Typhimurium ∆*tolR* (STm-GMMA), *S*. Typhimurium ∆*lon* ∆*tolR* (lon-GMMA), or *S*. Typhimurium ∆*bcsA* ∆*yihW* ∆*tolR* Tn10C (Tn10C-GMMA). Secondary and tertiary immunizations were performed with 5 µg of GMMAs at three-week intervals. Serum samples were collected on days 0, 21, 42 and 63.

### 2.11. Murine Infection Experiments

For competitive index (CI) experiments, two groups of six C57BL/6 mice were challenged with a mixed inoculum consisting of 1:1 ratio of Kan^R^ and Cam^R^ *S*. Typhimurium 14028 strains containing the sig70_16 *luxCDABE* construct [42]. Mice were challenged via oral gavage with a total CFU of ~10^7^.

Immunized C57BL/6 or BALB/C mice were challenged 21 days after the final immunization by oral gavage with 10^7^ CFU of Kan^R^ *S*. Typhimurium containing the sig70_16 *luxCDABE* construct.

Infected mice were weighed daily and monitored for clinical signs of infection. Mice that had a >20% drop in weight were humanely euthanized. All mice were humanely euthanized 4–7 days post-infection. Spleen, liver, mesenteric lymph nodes (MLN) and cecum were collected from each mouse. Blood was collected from C57BL/6 mice.

Collected organs were placed in a 2 mL Eppendorf Safe-Lock tube containing 1 mL of phosphate-buffered saline (PBS) and a 5 mm steel bead (Product# 69989; Qiagen, Hilden, Germany) and homogenized using a mixer mill (MM400; Retsch Gmbh, Haan, Germany) for 5 min at 30 Hz. Serial dilutions of organ homogenates and blood were plated on LB agar supplemented with 50 µg/mL Kan and the number of *Salmonella* CFU were enumerated. Organs collected from mice infected with a mixed inoculum were plated on both Kan and Cam agar. The CI values were calculated as follows: (CFU mutant/CFU wildtype)_output_/(CFU mutant/CFU wildtype)_input_.

### 2.12. ELISA

Serum IgG levels specific for CA, tgD, CRM197 and GMMAs were measured by ELISA. Briefly, 96-well plates were coated with CA (2 µg per well), tgD, CRM197 or GMMA (0.1 µg per well) in coating buffer at 4 °C overnight. Wells were blocked for 30 min at RT with 5% skim milk dissolved in Tris-buffered saline (TBS) containing 0.05% tween 20 (TBST). Sera were serially diluted in TBST, starting at 1:100 in 5-fold dilutions. Alkaline phosphatase-conjugated goat-anti-mouse IgG at a dilution of 1:1500 was used to detect bound IgG. The reaction was visualized with p-nitrophenyl phosphate, and absorbance was read at 405 nm with a reference wavelength of 409 nm.

### 2.13. Statistical Analysis

Statistical analyses were performed using GraphPad Prism version 8.0.0 for Mac OS (GraphPad Software, San Diego, CA, USA).

The normality of all datasets was assessed using the Shapiro–Wilk normality test. For luciferase assays, if the data were not normally distributed, comparisons were made using the Mann-Whitney test. If data were normally distributed, comparisons were made using the unpaired *t*-test.

For murine infections, if the data were normally distributed, comparisons were performed using unpaired *t*-test with Welch’s corrections. If any data sets were not normally distributed, comparisons were performed using the Mann–Whitney test.

For CI experiments, if the data were normally distributed, a one-sample *t*-test was used to determine if the mean was significantly different from one. If the data were not normally distributed, the Wilcoxon signed-rank test was used to determine if the median CI was significantly different from one.

## 3. Results

### 3.1. The yih Operons Are Not Involved in S. Typhimurium Virulence

**Figure 1:**
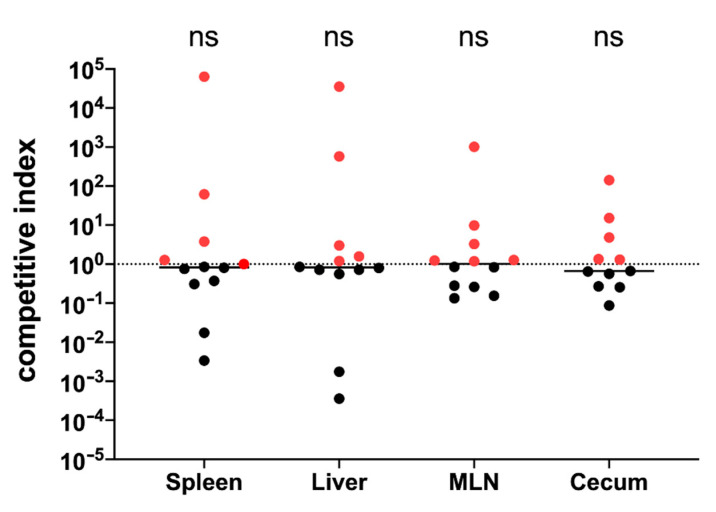
Evaluating the role of *yih* operon in the virulence of *S*. Typhimurium. A competitive index experiment was performed with C57BL/6 mice orally infected with wildtype and *S*. Typhimurium 14028 Δ*yih*. At 4–7 days post-infection, the CFU levels were enumerated from the liver, spleen, cecum and MLN. Each dot represents the CFU counts (per organ) from the designated organ from a single mouse. Competitive index values were calculated from each organ as follows: (CFU *yih* mutant/ wt)_output_/(CFU *yih*/wt)_input_. A CI value of 1, which represents a situation where both strains are equally virulent, is represented by the horizontal dotted line. Red circles represent CI values where the *S*. Typhimurium 14028 Δ*yih* strain won the competition. Statistical differences between groups of mice were noted as ns: *p* > 0.05.

The divergently transcribed *yihUTSRQPO* (*yihU*) and *yihVW* operons have been identified as the operons controlling O-Ag capsule biosynthesis in *Salmonella* [9]. Using bioluminescence, White et al. [43] showed that the *yihU* operon is expressed during infection. Since virulence factors are often optimal vaccine candidates, we wanted to determine if the *yih* operons are involved in *Salmonella* virulence. We performed a competitive index experiment in C57BL/6 mice orally challenged with a 1:1 mixture of *S*. Typhimurium 14028 (wildtype) and *S*. Typhimurium 14028Δ*yih*. At 4–7 days post-infection, mice were euthanized and bacterial loads in the spleen, liver, MLN and cecum were enumerated. An equal number of wildtype and *S*. Typhimurium 14028Δ*yih* were recovered from all organs tested (Figure 1), suggesting that the *yih* operons are not involved in *Salmonella* virulence.

### 3.2. YihW Represses Expression of the Yih Operons

One of the problems of commercializing CPS-based vaccines is the difficulty to produce large quantities of CPS at an industrial scale. Therefore, our first objective was to boost O-Ag capsule production in *Salmonella* serovar Typhimurium by increasing the expression of the *yih* operons. It is often difficult and inefficient to purify CPS in the presence of cellulose because it non-specifically binds the materials in the extracellular matrix [31]. Therefore, a cellulose-negative strain was used for *Salmonella* CPS purification.

**Figure 2:**
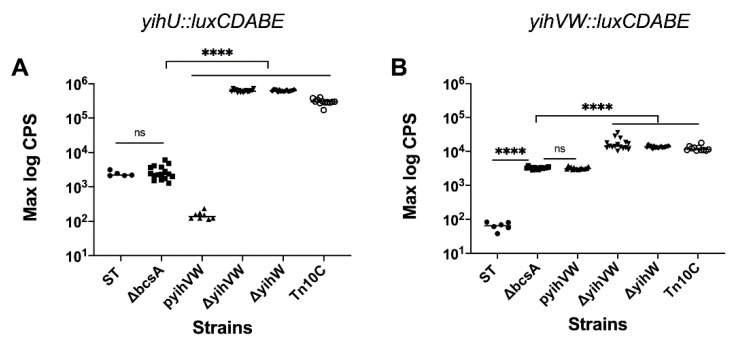
Expression of *yihUTSRQPO* and *yihVW* operons in *S.* Typhimurium. Promoter luciferase fusions for (**A**) *yihUTSRQPO* and (**B**) *yihVW* were used to measure expression in *S*. Typhimurium (ST, wildtype), *S*. Typhimurium Δ*bcsA* (ΔbcsA), *S*. Typhimurium Δ*bcsA* pBR322-y*ihVW* (pyihVW), *S*. Typhimurium Δ*bcsA* Δ*yihVW* (ΔyihVW), *S*. Typhimurium Δ*bcsA* Δ*yihW* (ΔyihW) and *S*. Typhimurium Δ*bcsA* Δ*yihW* Tn*10* (Tn10C). Cultures were grown in 1% tryptone at 28 °C with agitation, and luminescence (in counts per second (cps)) was recorded every 30 min for 48 hours. The log maximum CPS value recorded over 48 hours is shown. Statistical differences were noted as **** *p* < 0.0001, ns: *p* > 0.05.

Genes in the *yihUTSRQPO* operon are homologous to carbohydrate metabolism genes, while genes in the *yihVW* operon are homologous to regulatory genes (*yihV* have homology to kinases, while *yihW* has homology to glycerol-3-P regulon repressor) [9]. To determine if genes in the *yihVW* operon have a regulatory effect on the expression of the *yih* operons, *yihUTSRQPO* and *yihVW* promoter::*lux* fusions were used to measure the promoter activity of both operons in *S*. Typhimurium. Deletion of cellulose synthase (Δ*bcsA*) had no effect on the expression of *yihU* (Figure 2A), and increased *yihVW* expression by ~70-fold (Figure 2B). Plasmid-based overexpression of *yihVW* had no effect on *yihVW* expression but reduced *yihU* expression by ~20-fold. Deletion of *yihVW* or *yihW* alone increased *yihU* expression by ~100-fold (Figure 2A). Our results are consistent with YihW being a transcriptional repressor of the *yihUTSRQPO* operon.

To determine if increased expression from the *yihU* operon would lead to increased production, O-Ag capsule was purified from *S.* Typhimurium Δ*bcsA* and *S.* Typhimurium Δ*bcsA* Δ*yihW* strains. Two times more crude CPS was purified from *S.* Typhimurium Δ*bcsA* Δ*yihW* compared to *S.* Typhimurium Δ*bcsA*. To isolate O-Ag capsule from crude CPS, anion and size exchange chromatography were performed and O-Ag capsule containing fractions were identified using EPS-specific serum. Similar quantities of O-Ag capsule were purified from *S.* Typhimurium Δ*bcsA* and *S.* Typhimurium Δ*bcsA* Δ*yihW* after chromatography (Table 3), suggesting that the increase in *yihU* expression did not lead to increased production of O-Ag capsule.

### 3.3. Effect of Precursor Sugars on yihUTSRQPO Promoter Activity

**Figure 3:**
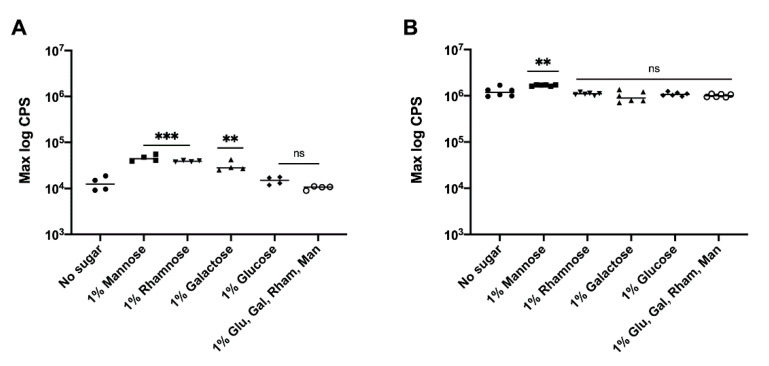
Effect of different precursor sugars on the expression of *yihUTSRQPO*. The *S*. Typhimurium Δ*bcsA* (**A**) and *S*. Typhimurium Δ*bcsA* Δ*yihW* (**B**) strains were grown in 1% tryptone media supplemented with or without different precursor sugars (glucose, galactose, rhamnose, mannose, or all four sugars), at 28 °C. Luminescence (in counts per second (cps)) was recorded every 30 min for 48 hours. The log maximum CPS value recorded over 48 hours is shown. Statistical differences were noted as ** *p* < 0.01, *** *p* < 0.001, or ns: *p* > 0.05.

The lack of adequate amounts of precursor sugars could be one possible explanation for why 100-fold increased expression of the *yihUTSRQPO* operon in *S.* Typhimurium Δ*bcsA* Δ*yihW* did not lead to increased production of O-Ag capsule. Hence, we wanted to determine if increased amounts of O-Ag capsule could be isolated from *S.* Typhimurium Δ*bcsA* Δ*yihW* grown in media supplemented with precursor sugars required for O-Ag capsule biosynthesis. *S*. Typhimurium O-Ag capsule is made up of tetrasaccharide repeating units of galactose, rhamnose, mannose and abequose, with abequose and galactose residues partially substituted with a glucose side chain [17]. Using a luciferase reporter assay, we examined the effect of precursor sugars on *yihU* expression. The addition of mannose, rhamnose and galactose to the growth media increased *yihU* expression approximately 5-fold in *S*. Typhimurium ∆*bcsA* (Figure 3A), while only the addition of mannose slightly increased *yihU* expression in *S*. Typhimurium ∆*bcsA* ∆*yihW* (Figure 3B). However, increased gene expression in the presence of precursor sugars had no measurable effect on O-Ag capsule production (data not shown). This indicated that the lack of increased O-Ag capsule production by *S*. Typhimurium ∆*bcsA* ∆*yihW* was not due to the absence of an adequate amount of precursor sugars.

### 3.4. Overproduction of CPS in Transposon Mutants

Since increased expression of the *yih* operons and supplementation of growth media with precursor sugars did not boost the amount of O-Ag capsule purified from *S*. Typhimurium ∆*bcsA* ∆*yihW*, we hypothesized that other regulators besides *yihW* may be negatively regulating the biosynthesis of O-Ag capsule. To identify unknown factors that negatively regulate CPS biosynthesis in *Salmonella*, random mutagenesis was performed in *S*. Typhimurium ∆*bcsA* ∆*yihW* using Tn*10*dtet transposon. It was expected that random mutagenesis in *S*. Typhimurium ∆*bcsA* ∆*yihW,* with ~100-fold increased expression of *yihU*, would lead to a mutant strain that overproduces O-Ag capsule.

Bacterial colonies overproducing CPS often have mucoid and watery surfaces that can be visually detected [11,44], hence colony morphology was used to identify CPS overproducing strains. After random mutagenesis, seven potential high-CPS-producing isolates with mucoid morphologies were identified (named Tn*10*A–G). Nested PCR was used to identify the genomic sites of Tn10 insertions (Table 3), and crude CPS was purified from each mutant. At least 2 times more crude CPS was isolated from Tn*10* C, D, E and G compared to *S*. Typhimurium ∆*bcsA* ∆*yihW* (Table 3). Following our standard O-Ag capsule purification procedure, ~10 times more material was obtained from mutant strain Tn*10*C, hence subsequent experiments were performed with this mutant. Using luciferase assays, we showed that *yihU* and *yihVW* expressions were similar in Tn*10*C and *S*. Typhimurium ∆*bcsA* ∆*yihW* (Figure 2), suggesting that increased *yihU* expression was not responsible for the increased amount of CPS produced in the Tn*10*C strain.

### 3.5. Increased CPS Production by Salmonella Grown in Buffered Media

O-Ag capsule was purified from *S.* Typhimurium grown in EPS media with a carbon to nitrogen ratio of 10:1. We reasoned that a drop in pH due to increased glucose metabolism may reduce growth and limit the amount of CPS produced by *S*. Typhimurium ∆*bcsA* ∆*yihW* Tn*10*C. Therefore, we investigated the effect of buffering the pH on CPS production. Approximately four times more crude CPS was isolated from strain Tn*10*C grown in EPS media buffered with MOPS compared to unbuffered media (Table 3).

### 3.6. Overproduction of Colanic Acid in S. *Typhimurium*

Since immune serum raised to whole *Salmonella* EPS [9] was used to guide the purification process, we performed monosaccharide composition analysis to characterize the final purified CPS. The amounts of fucose, galactose and glucose present in our crude and final purified CPS showed that colanic acid was purified from *S*. Typhimurium ∆*bcsA* ∆*yihW* Tn*10*C, and not O-Ag capsule (Table 4). Furthermore, detection of rhamnose and mannose suggests the presence of contaminating LPS in purified colanic acid.

WcaJ is a glycosylase that is part of the *wca* gene cluster important for the biosynthesis and transport of colanic acid in *Salmonella* [45]. To verify the overproduction of colanic acid, we generated a *S*. Typhimurium ∆*bcsA* ∆*yihW* Tn*10*C Δ*wcaJ* strain. Deletion of *wcaJ* resulted in the loss of the mucoid morphology associated with CPS overproduction (data not shown), further indicating that colanic acid was being over-produced from *S*. Typhimurium ∆*bcsA* ∆*yihW* Tn*10*C.

### 3.7. Removal of Contaminating LPS from Purified CPS

LPS is a common contaminant of purified CPS from Gram-negative bacteria, and since it is toxic in high amounts, the presence of LPS is not desirable in vaccine formulations [46]. Triton X-114 or acid hydrolysis with acetic acid are routinely used to remove LPS from purified CPS [38,39]. To determine the most efficient method for LPS elimination, purified colanic acid was treated with either Triton X-114 or acetic acid.

Triton X-114 is a non-ionic detergent that separates into detergent-rich and detergent-poor phases at room temperature [46]. When mixed with purified CPS, LPS preferentially goes into the detergent-rich phase, due to non-polar interactions between lipid A and Triton X-114 detergent. Several rounds of separation with Triton X-114 are usually performed to reduce the amount of LPS present in purified CPS [38]. We performed three rounds of LPS removal with Triton X-114, however increasing amounts of material were lost with each separation round. Only 50 mg of colanic acid was isolated from 1000 mg of crude CPS.

Following acid hydrolysis with acetic acid, the endotoxic portion of LPS, lipid A, is cleaved off the core oligosaccharide and forms precipitates which can be separated by centrifugation. After lipid A removal, colanic acid was purified using anion exchange and size exclusion chromatography. Approximately 200 mg of colanic acid was purified from 1000 mg of crude CPS.

After treatment with Triton X-114 or acid hydrolysis, the limulus amebocyte lysate (LAL) assay was used to quantify the amount of LPS (i.e., lipid A) present in purified colanic acid. About 4.9 × 10^4^ endotoxin unit (EU)/mL was present after three rounds of LPS removal with Triton X-114, while 1.4 × 10^4^ EU/mL of LPS was present after treatment with acetic acid followed by size exclusion chromatography. LPS at a concentration between 1 × 10^3^ and 4.55 × 10^6^ EU/mL has been shown to be safe when administered to a 20 g mouse [46,47]. Hence, both methods reduced LPS to a safe level, however, less material was lost when acid hydrolysis was used for LPS elimination.

### 3.8. Immune Response Induced by Colanic Acid

The objective of this study was to boost the amount of CPS purified from *S*. Typhimurium for immunization trials. Random mutagenesis with Tn*10*dtet transposon aimed at increasing the amount of O-Ag capsule purified from *S*. Typhimurium led to the overproduction of colanic acid. Colanic acid has previously been associated with survival in non-host environments [11,12], however since it is a conserved CPS, we wanted to determine if immunization with colanic acid could induce a protective immune response against *Salmonella*.

**Figure 4:**
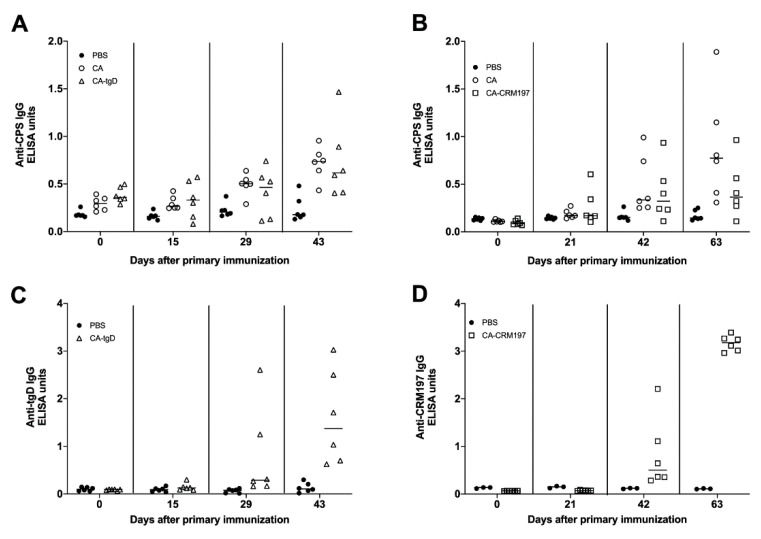
Immune response to colanic acid, truncated glycoprotein D and inactivated diphtheria toxin. ELISA was performed with serum collected from CB7BL/6 mice (**A**,**C**) or BALB/c mice (**B**,**D**), immunized with phosphate-buffered saline (PBS), colanic acid (CA), CA conjugated to inactivated diphtheria toxin (CA-CRM197) or CA conjugated to truncated glycoprotein D (CA-tgD).

Groups of six BALB/c or C57BL/6 mice were immunized intramuscularly with 50 µg of purified colanic acid formulated with TriAdj. TriAdj is a novel combination adjuvant platform comprised of (1) a TLR agonist, polyinosinic:polycytidylic acid (PolyI:C), (2) an immunostimulatory host defense peptide (HDP) and (3) polyphosphazene [48]. After primary immunization, two booster immunizations were administered at 21-day intervals. Immunization with colanic acid induced an anti-CPS acid IgG response in C57BL/6 (Figure 4A) and BALB/C (Figure 4B) mice.

To generate a specific and strong T-cell-dependent response, CPS are often conjugated to carrier proteins [49,50]. Colanic acid was conjugated to truncated bovine herpesvirus 1 glycoprotein D (tgD) [51,52] or the inactive form of diphtheria toxin (CRM197) [53]. In both cases, we detected high molecular weight material running above the size of the carrier proteins alone, indicating that successful conjugation had occurred (Supplementary Figure S1). C57BL/6 mice were immunized with 50 µg of colanic acid conjugated to CRM197 (CA-CRM197), while BALB/c mice were immunized with 1 µg of colanic acid conjugated to tgD (CA-tgD). Immunization with colanic acid or CA-CRM197 induced similar levels of anti-CPS IgG in C57BL/6 mice (Figure 4A). Immunization with colanic acid or CA-tgD also induced similar levels of anti-CA IgG in BALB/c mice (Figure 4B), however, mice were immunized with 50 times less CA-tgD.

Immunization with CA-CRM197 or CA-tgD induced robust anti-CRM197 IgG and anti-tgD IgG levels, respectively (Figure 4C,D). These results indicate that immunization with colanic acid alone or colanic acid conjugated to a carrier protein induced an immune response in mice.

### 3.9. Immunization with Colanic Acid Does Not Induce a Protective Immune Response

**Figure 5:**
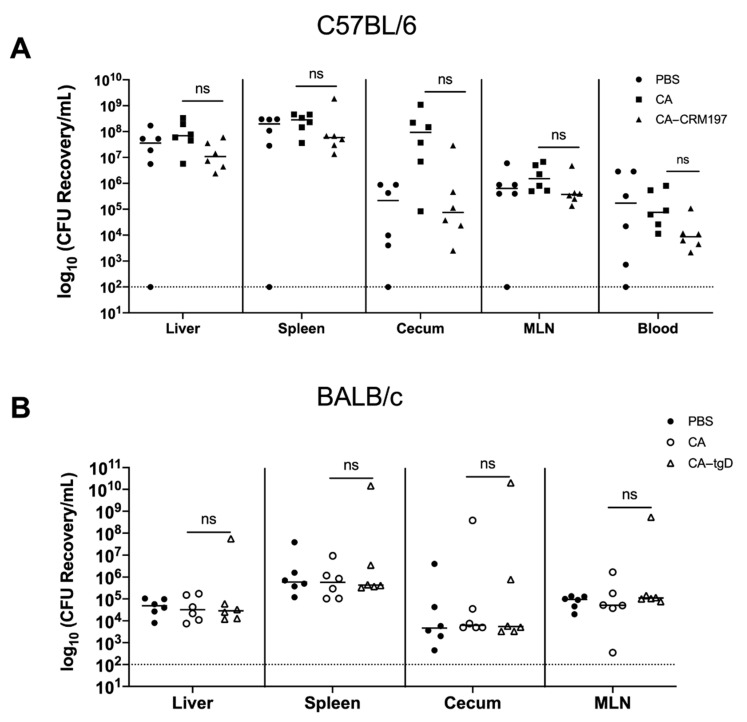
Amounts of *S*. Typhimurium recovered from mice previously immunized with colanic acid. (**A**) C57BL/6 mice were immunized with phosphate buffered saline (PBS), colanic acid (CA) or CA conjugated to inactivated diphtheria toxin (CA-CRM197). (**B**) BALB/c mice were immunized with PBS, CA or CA conjugated to truncated glycoprotein D (CA-tgD). All immunized mice were orally challenged with 10^7^ CFU of *Salmonella,* and 4–7 days post-infection, the liver, spleen, cecum, mesenteric lymph node (MLN) and blood (C57BL/6) were harvested. Organs were homogenized before plating on LB agar supplemented with kanamycin. The log_10_ CFU values of *Salmonella* recovered from individual organs from each mouse are shown. The dashed line represents the limit of detection of 100 CFU. For each group of mice, the black line represents the median values. Statistical significance: Not significant (ns): *p* > 0.05.

To determine if immunization with colanic acid or colanic acid conjugated to a carrier protein induced a protective immune response, mice were orally challenged with 10^7^ CFU of *S*. Typhimurium three weeks after the final immunizations. Four to seven days post-challenge, mice were euthanized, and liver, spleen, cecum, MLN or blood were collected for bacterial enumeration. Approximately 1-log less *Salmonella* was recovered from C57BL/6 mice immunized with CA-CRM197 compared to mice immunized with PBS or colanic acid (Figure 5A). No significant difference in *Salmonella* CFU levels was detected from BALB/c mice immunized with colanic acid, CA-tgD or PBS (control) (Figure 5B). These results indicated that an immune response induced by colanic acid alone or conjugated to tgD was not protective against future *Salmonella* infections.

### 3.10. Immunogenicity of GMMAs

GMMAs are targets for vaccine development because they are rich sources of outer membrane antigens that can often induce a strong immune response [20]. We wanted to determine if immunization with GMMAs purified from CPS overproducing isolates would induce a protective immune response against *Salmonella*. The deletion of *tolR* affects the stability of the linkage between the inner and outer membrane and results in increased GMMA production [54,55]. A *tolR* mutation has previously been linked to increased GMMA production in *Salmonella* [22], hence we purified GMMAs from colanic acid overproducing Tn*10*C with a *tolR* deletion (Tn*10*C GMMAs). As controls, we also purified GMMAs from *S*. Typhimurium Δ*tolR* (wildtype GMMAs) and *S*. Typhimurium Δ*tolR* Δ*lon* (Lon GMMAs). Lon is a cytoplasmic serine protease responsible for the proteolytic cleavage of several proteins, and *S. enterica* strains with *lon* mutations have been shown to overproduce colanic acid [56].

**Figure 6:**
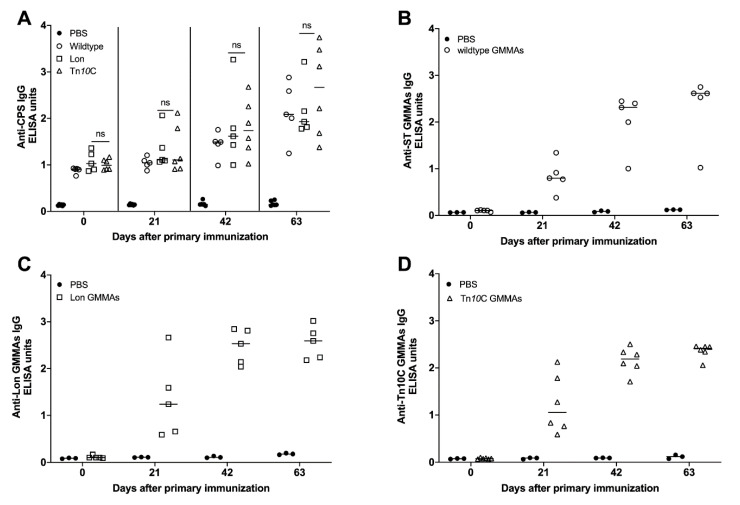
Immune response to GMMAs and colanic acid in immunized mice. Mice were immunized with GMMAs purified from *S*. Typhimurium 14028 ∆*tolR* (wildtype), *S*. Typhimurium 14028 ∆*tolR* ∆*lon* (Lon), Tn*10*C ∆*tolR* (Tn*10*C) or PBS. ELISA was performed with sera collected on days 0, 21, 42 and 63 and used to detect Anti-CPS IgG (**A**), Anti- *S*T GMMAs IgG (wildtype) (**B**), Anti-Lon GMMAs IgG (**C**) and Anti-Tn*10*C GMMAs IgG (**D**). Each point represents the value from an individual mouse, the horizontal line represents the median from each group of mice. Statistical significance: ns: *p* > 0.05.

Groups of six C57BL/6 mice were immunized intramuscularly with 50 µg of GMMAs purified from each strain. Two booster immunizations of 5 µg were given to each mouse at 3-week intervals. Serum from immunized mice was screened for the presence of antibodies that were specific to GMMAs and CPS. Immunization with GMMAs purified from wildtype, Δ*lon* or Tn*10*C strains induced a significant amount of anti-CPS antibodies 42 days after primary immunization (Figure 6A). There were no statistically significant differences between the amount of anti-CPS IgG induced by wildtype, Lon and Tn*10*C GMMAs.

Immunization with wildtype, Lon or Tn*10*C GMMAs induced increasing amounts of anti-GMMAs IgG on days 21, 42 and 63 (Figure 6B–D). Our results indicate that immunization with GMMAs induces CPS- and GMMA-specific immune responses.

### 3.11. Immunization with GMMA Reduced Salmonella Colonization of Mice Organs

To assess the level of protection induced by GMMAs, three weeks after the final immunizations, mice were orally challenged with 10^7^ CFU of *S*. Typhimurium. All mice were euthanized 4–7 days after challenge and protection was assessed by the levels of *S*. Typhimurium colonization in the liver, spleen, cecum, MLN and in blood samples.

**Figure 7:**
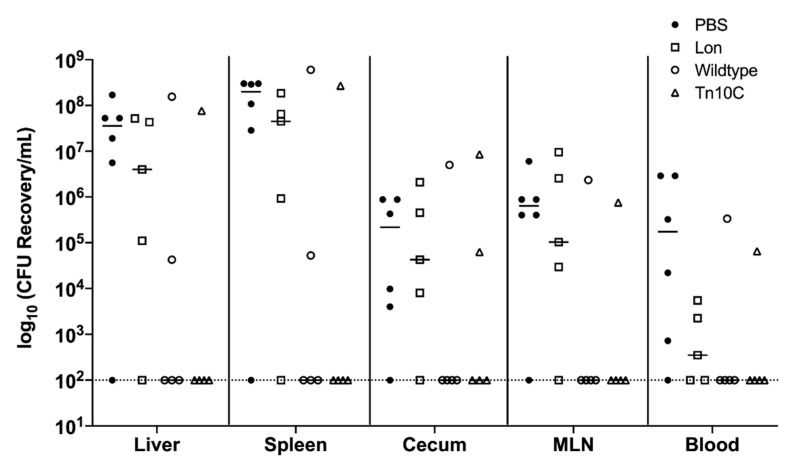
Ability of GMMAs to protect mice against lethal challenge of *S*. Typhimurium. C57BL/6 mice were immunized with PBS and GMMAs purified from *S*. Typhimurium Δ*tolR* (wildtype), *S*. Typhimurium Δ*tolR* Δ*lon* (Lon) and *S*. Typhimurium Δ*tolR* Δ*bcsA* Δ*yihW* Tn*10*dtet (Tn*10*C). Immunized mice were orally challenged with 10^7^ CFU of *S*. Typhimurium 14028, and 4–7 days post-infection, mice were euthanized, and liver, spleen, cecum, MLN and blood were collected for bacterial enumeration. The log_10_ CFU of *Salmonella* recovered from each mouse is shown. The dotted line represents the limit of detection of 100 CFU. The black line represents the median log CFU values determined from each group of mice.

*S.* Typhimurium was not recovered from 3 of 5 mice immunized with wildtype GMMAs, and 4 of 5 mice immunized with Tn*10*C GMMAs, whereas 4 of 5 mice immunized with Lon GMMAs and 5 of 6 non-immunized control mice had significant levels of *S*. Typhimurium colonization (Figure 7). Based on these results, we hypothesize that immunization with wildtype and Tn*10*C GMMAs induced a partially protective immune response against *S*. Typhimurium.

Mice immunized with wildtype or Tn*10*C GMMAs showed ~5-log reduction of bacterial colonization in the liver and spleen, and ~3-log reduction in the cecum, MLN and blood, compared to control mice (Figure 7). Mice immunized with Lon GMMAs showed a 1-log reduction in the liver, spleen, cecum and MLN and a 4-log reduction in the blood compared to control mice (Figure 7). Our results indicate that immunization with GMMAs reduces bacterial colonization of mice organs.

## 4. Discussion

Immune responses directed against CPS form the basis for some of the most successful human vaccines. CPS conjugated to carrier proteins are the basis for most CPS-based vaccines currently licensed for use, against human pathogens such as *Haemophilius influenzae* type b, *Neisseria meningitidis*, *Streptococcus pneumoniae* and *Salmonella* serovar Typhi [57,58,59]. There is currently no licensed vaccine against NTS. Due to the success of Vi CPS-based vaccines against typhoid fever [60,61], there is renewed interest in developing a CPS-based vaccine against NTS serovars that cause gastroenteritis and invasive blood stream infections.

Colanic acid is a common CPS mainly associated with survival of *Salmonella* cells in adverse environmental conditions [12]. However, it has been shown to contribute to *Salmonella* biofilm formation on mammalian cell lines and chicken intestinal epithelium [61]. The role of SrfA in CPS biosynthesis is unknown; however, insertional inactivation of *srfA* led to increased colanic acid production. SrfA is a virulence effector involved in modulating the host inflammatory response by promoting the activation of NF-κB signaling [62,63]. Since colanic acid may be produced in vivo during infection, we wanted to determine if a colanic acid-specific immune response could provide protection against lethal *S*. Typhimurium challenge. Similar CFU of *Salmonella* were recovered from mice immunized with colanic acid or PBS (control). *Salmonella* overproducing colanic acid has previously been shown to be susceptible to the bactericidal activity of human serum and to phagocyte-mediated killing [44,64]. Based on these results, we conclude that immunization with colanic acid does not induce a protective immune response. Although similar CFU values of *S.* Typhimurium were recovered from the organs of CA-tgD- and PBS-immunized mice, the reduced CFU recovered from mice immunized with CA-CRM197 suggests that colanic acid conjugated to CRM197 can induce a partially protective immune response in mice.

GMMAs are attractive vaccine candidates because they are comprised of multiple antigens that can stimulate an immune response [65]. Several studies have focused on developing GMMAs purified from bacteria with different genetic modifications as vaccine candidates for NTS [29]. GMMAs purified from *S*. Typhimurium with truncated LPS or deficient in flagellin production have been shown to induce cross-reactive antibody responses and cross-protection against *Salmonella* serovar Choleraesuis and Enteritidis [66,67]. Our results showed that GMMAs purified from colanic overproducing *Salmonella* reduced bacteria colonization in mice organs. Although Tn*10*C and Lon GMMAs were isolated from colanic acid overproducing strains, immunization with Tn10C GMMAs reduced bacterial colonization to a greater extent compared to Lon GMMAs. This result suggested the presence of different antigens on Tn10C GMMAs as compared to Lon-GMMAs. This conclusion is supported by several studies that have shown that the composition of GMMAs can vary with regards to growth phase, growth condition and genetic mutation present in bacteria [68,69,70]. Based on our results, we propose that engineered GMMAs with high CPS content should be developed as vaccine candidates. LPS-OAg and GMMAs alone have been shown to induce protective immune responses against NTS in mouse models of infection [14,29]. However, none of these antigens have been successfully developed as a vaccine against NTS. Since GMMAs indue a more diverse immune response [70], and LPS-OAg is the target of protective immune response against NTS [14,15,16], we speculate that GMMAs with high concentrations of LPS-OAg will induce a diverse and cross-protective immune response against NTS.

The *S. enterica* O-Ag capsule was thought to be a potential vaccine candidate because it has similar repeating units as LPS O-Ag, which is the target of protective immunity against NTS [14,15,16]. Gibson et al. [9] speculated that precursor sugars synthesized by the LPS O-Ag machinery are modified, assembled and translocated out of the cell as O-Ag capsules by gene products from the divergent *yihUTSRQPO* and *yihVW* operons [9]. The increased expression of *yihUTSRQPO* in a Δ*yihW* mutant is consistent with YihW being a repressor of the *yih* operons. The repressive effect of YihW has also been described in *E. coli,* where three *yihW* binding sites were identified in the *yih* promoter region [71,72]. Overexpression of genes often leads to the overproduction of specific proteins or CPS. However, a 100-fold increased expression of the *yihUTSRQPO* operon in the Δ*yihW* mutant did not result in increased production of O-Ag capsule. The introduction of precursor sugars into growth media often leads to increased CPS biosynthesis [73,74,75]. However, O-Ag capsule biosynthesis was not increased in the presence of excess precursor sugars. In addition, random mutagenesis aimed at increasing the amount of O-Ag capsule produced by *S*. Typhimurium Δ*bcsA* Δ*yihW* led to increased colanic acid production. Based on these results, we speculate that the *yih* operons are not involved in biosynthesis of the O-Ag capsule.

Using enzymatic assays with purified proteins and mass spectrometric analysis of products, genes in the *yih* operons have been shown to be required for the catabolism of sulphoquinovose (SQ) by *E. coli* [76,77]. SQ is a major constituent of the human diet and about 10 pentagrams are produced by photosynthetic organisms annually [78]. Analysis of *Salmonella* mutants indicated that genes in the *yih* operons are involved in the colonization and persistence of *Salmonella* on plant surfaces [79,80], biofilm formation [81], resistance to desiccation [9] and resistance to killing by human immune serum [13]. Due to the high nucleotide (80%) and protein (80–92%) sequence identity with the *E. coli* operon, we speculate that the *yih* operons are also involved in SQ catabolism by *S. enterica*. However, due to the phenotypes associated with mutant strains, there is a possibility that genes in the *yih* operons have dual functionality. In *Salmonella,* the *yih* operons may be involved in SQ catabolism and in the synthesis of an unknown CPS or the modification of the antigenic content of the outer membrane. The serum used by Gibson et al. [9] for the initial identification of the *yih* operons was generated against whole *Salmonella* EPS fraction and was found to be cross-reactive to an uncharacterized CPS [9]. This might explain the lack of CPS in *S*. Typhimurium Δ*yihO* observed by Marshall and Gunn [13] using confocal microscopy [9,13]. A role for the *yih* operons in the modification of the antigenic content of the outer membrane provides an explanation for the exclusive production of phase 1 flagellin FliC by *S*. Typhimurium Δ*yih* mutants, and the production of ~25% to 45% more short LPS (with 1 to 8 LPS O-Ag repeating units) by *S*. Typhimurium Δ*yih* mutants compared to wildtype [13]. More research is required to clarify the role of the *yih* operons in *Salmonella*.

Based on our research, we predict that the O-Ag capsule described by Gibson et al. [9] could represent very long-chain LPS O-Ag, which would provide an additional explanation for why increased expression of the *yihU* operon did not lead to increased O-Ag capsule production. *S*. Typhimurium has a tri-modal distribution of LPS due to the varying length of the O-Ag repeating units. Short LPS O-Ag (S-O-Ag) comprised of 1 to 15 repeating units, long LPS O-Ag (L-O-Ag) composed of 16 to 35 repeating units and very long LPS O-Ag (VL-O-Ag) comprised of more than 100 repeating units [82,83,84]. We speculate that the O-Ag capsule and VL-O-Ag could be the same CPS because they both have similar banding patterns on SDS-PAGE [9,13], are composed of similar tetrasaccharide repeat units [17] and have been implicated in similar functions [13,83]. Although the O-Ag capsule was found to be glycosylated at the tyvelose and galactose residues, high molecular weight LPS with varying glycosylation levels has been previously described in *Salmonella* [84]. Hence, the O-Ag capsule may be VL-O-Ag glycosylated at the tyvelose and galactose sugars. More research is needed to determine the structure of this polysaccharide and others that are present on the cell surface of *S. enterica* isolates.

## 5. Conclusions

Although CPS has been the focus of subunit vaccine development against NTS, none have been tested in clinical trials. The immune response to LPS-O-Ag is strain-specific, while colanic acid does not induce a protective immune response. Research on CPS-based vaccines against NTS will have to focus on identifying novel CPS that can be developed as vaccines. Currently, GMMAs may be the best strategy for vaccine development, because they are highly immunogenic, deliver multiple antigens in their natural context and can induce a cross-protective immune response. With the rise in multi-drug-resistant isolates and the increasing incidence of invasive bloodstream infections, a vaccine is urgently needed to reduce the prevalence of NTS infections.

## Figures and Tables

**Table 1:** Strains and plasmids used in this study.

Strains or Plasmids	Genotype	Source or Reference
Strains		
*S*. Typhimurium LT2		
TT10423	*proAB47*/F’ *pro*(+) *lac*(+) *zzf*-1831::*Tn10*(del) 16 (del) 17	[30]
*S*. Typhimurium 14028	Wild-type strain	ATCC
Δ*bcsA*	Deletion of *bcsA* ORF	[31]
Δ*yihVW*	Deletion of *yihVW* operon	This study
Δ*yihW*	Deletion of *yihW* ORF	This study
Δ*yih*	Deletion of *yihUTSRQPO* and *yihVW* operons	This study
Δ*bcsA* Δ*yihW*	Deletion of *bcsA* and *yihW* ORF	This study
Δ*wcaJ*	Deletion of *wcaJ* ORF	This study
Tn*10*A	Tn10dtet insertion in *Tnp IS200*	This study
Tn*10*B	Tn10dtet insertion in *filD*	This study
Tn*10*C	Tn10dtet insertion in *srfA*	This study
Tn*10*D	Tn10dtet insertion in *stm14_2260*	This study
Tn*10*E	Tn*10*dtet insertion in *fhlA*	This study
Tn*10*F	Tn10dtet insertion in o*mpS*	This study
Tn*10*G	Tn10dtet insertion in *stm14_3662*	This study
Tn*10*C Δ*tolR*	Tn10dtet insertion in *srfA* and deletion of *tolR* ORF	This study
Δ*tolR*	Deletion of *tolR* ORF	This study
Δ*lon* Δ*tolR*	Deletion of *lon* and *tolR* ORF	This study
Plasmids		
pNK972	pBR322 derived plasmid with Tn10 transposase gene	[32]
pBR322/*yihVW*	*yihVW* from *S*. Typhimurium 14028	This study
pCS26	Bacterial luciferase	[33]
pCS26-*yihUTSRQPO*	*yihU* promoter	[9]
pCS26-*yihVW*	*yihVW* promoter	[9]

**Table 2:** Oligonucleotides used in this study.

Primer	Sequence (5′–3′)	Purpose
yih operon F	TTATTGGCCGGATAAAGCGCTGACGCGACC CTCCGGCGCAAGGGCGCTTGGTGTAGGCTG GAGCTGCTTC	To amplify *cat* gene from pKD3 to generate Δ*yih* strain by lambda-red recombination
yih operon R	AATATAGGGAAGCCGCCATCCATCGGGATG GATAAAGCGGCAAGCGTCGTCCTCCTTAGT TCCTATTCCG	
yih operon PF	GGTTATAGGCCTCACGGTTT	To confirm deletion of *yihUTSRQPO*/*yihVW* operons
yih operon PR	TAATACGCGGTTAAAGTCGATGT	
yihVWkoFOR	TTCGTGAAATTAAAATGAGCACATCGAAAATGCTTGAGGAATGACCATGGGTGTAGGCTGGAGCTGCTTC	To amplify *cat* gene product from pKD3 to generate Δ*yihVW* strain by lambda-red recombination
yihVWkoREV	TTGGCCGGATAAAGCGCTGACGCGACCCTCCGGCGCAAGGGCGCTTGTCACCTCCTTAGTTCCTATTCCG	
yihWkoFOR	TAATATGAGCAGTAGGAAGCTTTTAGAGGAATGCTCATGAGTGTAGGCTGGAGCTGCTTC	Used with yihVWkoREV to generate Δ*yihW* strain
yihVWdetect1	GCACATCGAAAATGCTTGAGGA	To confirm the deletion of *yihVW* and *yihW* from *S*. Typhimurium 14028
yihVWdetect2	ATATCGCCTGCATCACAGCG	
yihVWFOR	CGCGCTGCAGCTGTTTGTGATCGTATTTGTAATTTAT	Used to amplify *yihVW* from *S*. Typhimurium 14028 for cloning into pBR322.
yihVWREV	GATCGACGTCGCATCACAGCGCCGTTTTATTG	
yihVWseqF	GATCTTGCCGGGAAGCTAGAGTAAG	To confirm the cloning of *yihVW* into pBR322
yihVWseqR	GATCTTCTTGAAGACGAAAGGGCCT	
TL	TCCATTGCTGTTGACAAAGGGAAT	Used for nested PCR (first reaction) to identify the site of Tn*10*dtet insertion
TR	ACCTTTGGTCACCAACGCTTTTCC	
ARB1	GGCCACGCGTCGANNNNNNNNGATAT	
ARB6	GGCCACGCGTCGANNNNNNNNACGCC	
Universal Tn	GACAAGATGTGTATCCACCTTAAC	Used for nested PCR (second reaction)
ARB2	GGCCACGCGTCGACTAGTAC	
lonF	CTATACTATCTGATTACCTGGCGGACACTAAACTAAGAGAGAGCTCTATGATTCCGGGGATCCGTCGACC	To amplify *kan* gene product from pKD13 to generate Δ*lon* strain by lambda-red recombination
lonR	TTATTAGCGCTATTTGCGCGAGGTCACTATTTTGCGGTTACAACCTGCATTGTAGGCTGGAGCTGCTTCG	
lonPF	AACACGCCGTTGAATGTGTG	To confirm the deletion of *lon* from *S*. Typhimurium 14028
lonPR	TTATATCAGGCCTGCCACGC	
wcaJ-ko-F	ATCTCCCCTTACCGCCTGCGGGTAAGGGGCC AATCACAGGAACAACGATGATTCCGGGGATC CGTCGACC	To amplify *kan* gene product from pKD13 to generate Δ*wcaJ* strain by lambda-red recombination
wcaJ-ko-R	GTAAAATAGCCTTGTGGGTCAGGTTCTTAATA CGCCGCTTTATTAACAAATGTAGGCTGGAGCTGCTTCG	
wcaJ-verF	CCAGAACCTGTTCACAAGGC	To confirm the deletion of *wcaJ* from *S*. Typhimurium 14028
wcaJ-verR	GCCTGAATGTGGAATCACGC	
TolR-ko_For	TTCTGCACCGCCAGGCGTTTACCGTAAGCGA AAGCAACAAGGGGTAAGCCGTGTAGGCTGGAGCTGCTTC	To amplify *cat* gene product from pDK3 to generate Δ*tolR* strain by lambda-red recombination
TolR-ko_Rev	AAACTGTTCGCCTGTTACTCGCCGTCTTTCAAGCCAACGGGACGCAGACTCCTCCTTAGTTCCTATTCCG	
TolR-F	CTGCTCGACGTACTGTTG	To confirm the deletion of *tolR* from *S*. Typhimurium 14028
TolR-R	ATCACCTGTTCAGACGGCAG	

**Table 3:** Purification of CPS from *S*. Typhimurium wildtype and transposon mutants.

*S*. Typhimurium Strains ^A^	Identified Site of Tn10 Insertion	Crude Polysaccharide ^B^ (mg)	Polysaccharide after Chromatography (mg)	Endotoxin Removal with Triton X-114 (mg)
Δ*bcsA*	-	59.5	12.5	<0.5
Δ*bcsA* Δ*yihW*		130	18	<0.5
Tn*10*A	*Tnp IS200*	132	41.1	<0.5
Tn*10*B	*fliD*	44	-	-
Tn*10*C	*srfA*	500	157.2	10
Tn*10*D	*stm14_2260*	209	37	<2
Tn*10*E	*fhlA*	332	49.8	<0.5
Tn*10*F	*ompS*	67	-	-
Tn*10*G	*stm14_3662*	344	61	<0.5
Tn*10*C (MOPS) ^C^	-	2000	500	100

^A^ Tn*10*A-G are Δ*bcsA* Δ*yihW* mutant strains with Tn*10*Tet transposons inserted in the genome. ^B^ The amount of polysaccharide purified from 50 EPS agar plates is shown. ^C^ Tn*10*C (MOPS) represents strain Tn*10*C grown in EPS agar buffered with 40 mM MOPS.

**Table 4:** Monosaccharide composition (Mol%) of crude and final purified CPS from *Salmonella.*

Monosaccharides	Crude CPS	Polysaccharide Isolated from Crude CPS	Colanic Acid	O-Ag Capsule
Rhamnose	3.6 ± 0.3	1.5 ± 0.1	NA	22
Fucose	32.3 ± 0.9	31.9 ± 0.3	27	NA
Mannose	5.7 ± 0.2	8.4 ± 0.1	NA	24
Galactose	35.2 ± 0.4	34.6 ± 0.4	28.8	28
Abequose	NT	NT	NA	18
Glucose	22.4 ± 0.5	22.4 ± 0.3	17.9	9.1

Note: Experiments were conducted in triplicate. Sum of numbers presented in a column may not precisely be 100.0 due to rounding. NA: not applicable, NT: not tested. Reference for O-Ag capsule composition: Snyder et al. [17], colanic acid composition: Sutherland [8].

## Data Availability

The data presented in this study are available on request from the corresponding author.

## Supplementary Materials

The following are available online at https://www.mdpi.com/2076-393X/9/2/165/s1, Figure S1: Conjugation of S. Typhimurium CPS to carrier proteins. A,B. Western blots of truncated glycoprotein (tGD) conjugated to CPS, using tGD-specific immune serum (A) or CPS-specific immune serum (B) as the primary antibody. The same samples were loaded: tGD conjugated to itself (Lane 2), tGD alone (Lane 3) or tGD conjugated to CPS (Lane 4). Pre-stained protein ladder was loaded in Lane 1; the molecular weight of tGD was 66 kDa. C. SDS-PAGE analysis showing inactivated diphtheria toxin (CRM197) conjugated to CPS (Lanes 1 and 2), pre-stained protein ladder (Lane 3), and CRM197 alone (Lane 4). The molecular weight of CRM197 was 58 kDa.
