# Supplementary material for: A GMMA-CPS-Based Vaccine for Non-Typhoidal Salmonella
Source: Vaccines (Basel). 2021 Feb 17;9(2):165. doi: 10.3390/vaccines9020165 (PMC7922415; doi:10.3390/vaccines9020165)
Supplement: Supplementary file 1 [file vaccines-09-00165-s001.pdf]

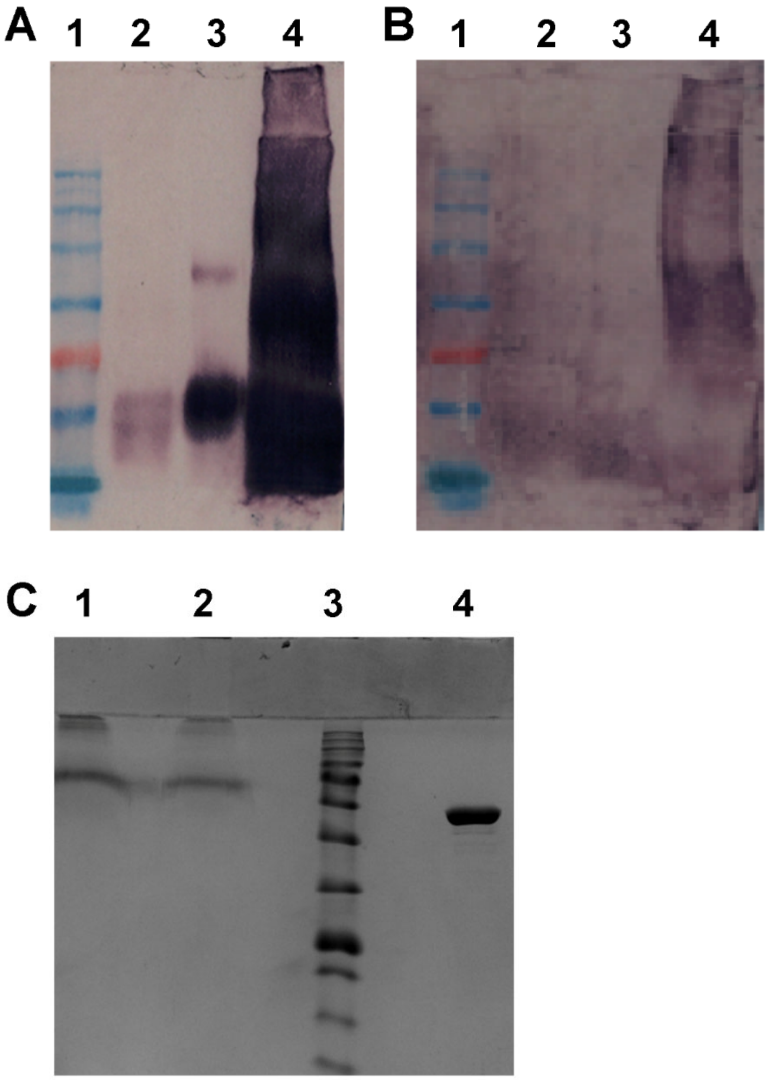

Supplementary Figure 1. Conjugation of *S*. Typhimurium CPS to carrier proteins.

A,B. Western blots of truncated glycoprotein (tGD) conjugated to CPS, using tGD-specific immune serum (**A**) or CPS-specific immune serum (**B**) as the primary antibody. The same samples were loaded: tGD conjugated to itself (Lane 2), tGD alone (Lane 3) or tGD conjugated to CPS (Lane 4). Pre-stained protein ladder was loaded in Lane 1; the molecular weight of tGD was 66 kDa.

C. SDS-PAGE analysis showing inactivated diphtheria toxin (CRM197) conjugated to CPS (Lanes 1 and 2), pre-stained protein ladder (Lane 3), and CRM197 alone (Lane 4). The molecular weight of CRM197 was 58 kDa.
